# Solvothermal synthesis of CdIn_2_S_4_ photocatalyst for selective photosynthesis of organic aromatic compounds under visible light

**DOI:** 10.1038/s41598-017-00055-5

**Published:** 2017-02-09

**Authors:** Cancan Ling, Xiangju Ye, Jinghu Zhang, Jinfeng Zhang, Sujuan Zhang, Sugang Meng, Xianliang Fu, Shifu Chen

**Affiliations:** 1 Department of Chemistry, Huaibei Normal University, Anhui Huaibei, 235000 People’s Republic of China; 2 Department of Chemistry, University of Science and Technology of Anhui, Anhui Fengyang, 233100 People’s Republic of China

## Abstract

Ternary chalcogenide semiconductor, cadmium indium sulfide (CdIn_2_S_4_), was prepared by a simple solvothermal method using ethylene glycol as a solvent, as well as indium chloride tetrahydrate (InCl_3_^.^4H_2_O), cadmium nitrate tetrahydrate [Cd(NO_3_)_2_^.^4H_2_O], and thiacetamide (TAA) as precursors. The resulted sample was subject to a series of characterizations. It is the first time to use CdIn_2_S_4_ sample as a visible light-driven photocatalyst for simultaneous selective redox transformation of organic aromatic compounds. The results indicate that the as-synthesized CdIn_2_S_4_ photocatalyst not only has excellent photocatalytic performance compared with pure In_2_S_3_ and CdS for the selective oxidation of aromatic alcohols in an oxygen environment, but also shows high photocatalytic redox activities under nitrogen atmosphere. A possible mechanism for the photocatalytic redox reaction in the coupled system was proposed. It is hoped that our current work could extend the applications of CdIn_2_S_4_ photocatalyst and provide new insights for selective transformations of organic compounds.

## Introduction

The photocatalytic technology has attracted much attention for the scholars of different fields since Fujishima and Honda found that TiO_2_ as electrode can decompose water to produce hydrogen and oxygen in 1972^1^. With the development of photocatalytic technology, current research work mainly focuses on the degradation of organic pollutants^2–4^, hydrogen production in water^5^, reduction of carbon dioxide^6^, selective oxidation of different alcohols^7^, and other kinds of organic transformation reactions^8–10^. Among them, selective oxidation of alcohols to corresponding aldehydes without further oxidation to carboxylic acids and carbon dioxide has become a research hotspot recently^11, 12^. This is because that the selective oxidation of aromatic alcohols into corresponding aldehydes is one of the most fundamental reactions in organic synthesis and fine chemical industry^13^. Traditional oxidants and reductants, such as KMnO_4_, ClO^−^, Fe, and Pt used in the redox process generally have toxicity, corrosivity, or preciousness. Additionally, these reaction systems are not only quite complicated, but also produce a lot of wastes. Therefore, one of the challenges as well as opportunities faced by the researchers is to develop a high-efficienct and environmental friendly method for selective organic transformations under mild conditions^14^.

It has been reported that semiconductor photocatalysts can be applied in the selective oxidation of alcohols to aldehydes rather than CO_2_ and H_2_O in the final products^15^. For example, Xu’s group reported that CdS was introduced as an efficient visible light photocatalyst for selective oxidation of saturated primary C-H bonds under ambient conditions^16^. Panda’s group found efficient photocatalytic selective nitro-reduction and C-H bond oxidation over ultrathin sheet mediated CdS flowers^17^. Wang and his coworkers reported molecular and textural engineering of conjugated carbon nitride catalysts for selective oxidation of alcohols with visible light^18^. In addition, other kinds of photocatalysts, such as noble metal-TiO_2_, Bi_12_O_17_Cl_2_ and boron nitride (BN)-sulfuret also exhibited relatively high efficiency for photosynthesis of organic compounds under visible light irradiation^19, 20^. However, most of the previously studied photocatalysts for the photocatalytic organic synthesis are composite materials or the monomer photocatalysts that are inefficient in most cases. In order to improve the photocatalytic activities, heterojunction photocatalysts, graphene and hexagonal NB-photocatalysts and cocatalyst-photocatalysts were often used for facilitating the separation of photoexcited electron-hole pairs and consequently improved the photocatalytic activities of the main catalysts^21, 22^. Unfortunately, the photocatalytic performance of the above photocatalysts still does not achieve a satisfactory result. Thus, how to gain higher photocatalytic activities of monomer catalysts which are easy to be synthesized and have highly efficient photocatalytic performance in the redox reaction under mild conditions, is still a constantly pursuing goal for researchers.

It is known that the selective oxidation of aromatic alcohols to corresponding aldehydes and reduction of nitrobenzene into aniline are the fundamental and significant reactions in commercial applications, because aromatic aldehydes, aniline, and their derivatives are important intermediates, which are widely used in pharmaceuticals, perfumes, manufacturing dye and other fine chemicals. It is also known that in the process of photocatalytic reaction, photoexcited holes (h^+^) and electrons (e^−^) are the active species in an inert gas atmosphere. And, the separation, migration and recombination of the photogenerated charge carriers are the major factors that influence the photocatalytic efficiency of photocatalysts^23–25^. How to develop an efficient photocatalyst which can take full advantage of the active species and promote the separation and transfer efficiency of the photo-induced charge carriers in one reaction system is still a hot topic. Recently, our group found that the selective oxidation of benzyl alcohol into benzaldehyde and the reduction of nitrobenzene into aniline under visible light irradiation in a system can be realized using CdS/g-C_3_N_4_ composite as a photocatalyst^26^. The photocatalytic activity for benzyl alcohol into benzaldehyde and reduction of nitrobenzene into aniline is mainly derived from the monomer CdS. Therefore, developing a novel monomer catalyst which is easier to be synthesized and has more efficient photocatalytic activity in the redox reaction is still an extremely important chemical challenge.

In this paper, CdIn_2_S_4_ photocatalyst prepared by a simple solvothermal method shows high photocatalytic activity for selective oxidation of benzyl alcohol into benzaldehyde and reduction of nitrobenzene into aniline in a coupled system. The as-prepared photocatalysts were characterized in detail by a series of technologies. Compared with pure CdS and In_2_S_3_, the as-synthesized CdIn_2_S_4_ photocatalyst exhibits a terrific activity for oxidation of benzyl alcohol into benzaldehyde in oxygen atmosphere under visible light irradiation, as well as selective oxidation of different aromatic alcohols into corresponding aldehydes and reduction of nitrobenzene into aniline in nitrogen atmosphere. A probable mechanism for the photocatalytic redox system of organic compounds was proposed. The superior photocatalytic performance of CdIn_2_S_4_ photocatalyst may be due to its suitable conduction band (CB) and valence band (VB) positions, higher surface area, and highly effective separation of photogenerated charge carriers. As far as we know, it is the first time to use CdIn_2_S_4_ sample as a visible light-driven photocatalyst for simultaneous selective redox transformation of organics in a coupled system.

## Results and Discussion

### Electronic properties

**Figure 1:**
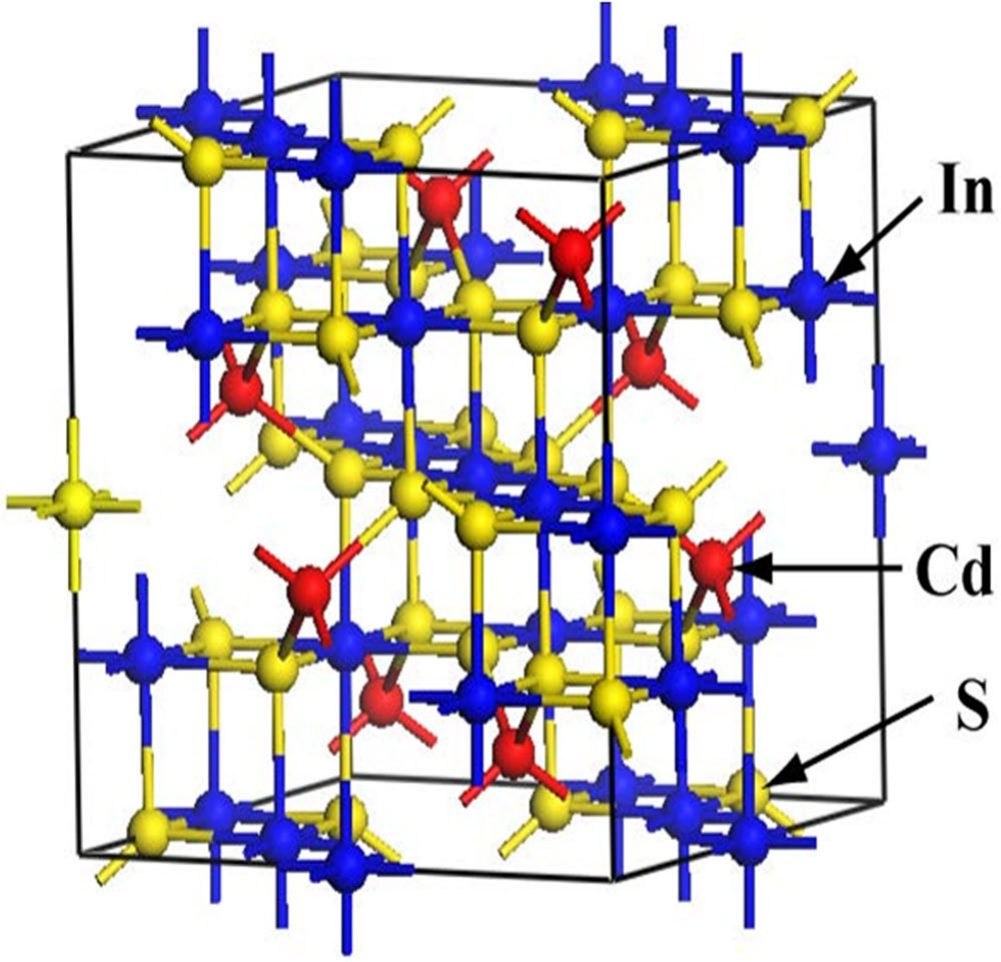
The conventional unit cell of CdIn_2_S_4_. (The Cd atom is denoted by the red sphere, In atom by the blue sphere, and S atom by the yellow sphere).

The conventional unit cell of CdIn_2_S_4_ is shown in Fig. 1.The lattice parameters (a = b = c = 10.845 Å, and α = β = γ = 90°) were taken from the XRD data and optimization results were performed by DFT before calculating electronic properties. The optimized lattice constants are a = b = c = 10.622 Å and α = β = γ = 90°, and the deviations between the calculated and experimental values are within 2.0%, indicating that the DFT calculation is accurate.

**Figure 2:**
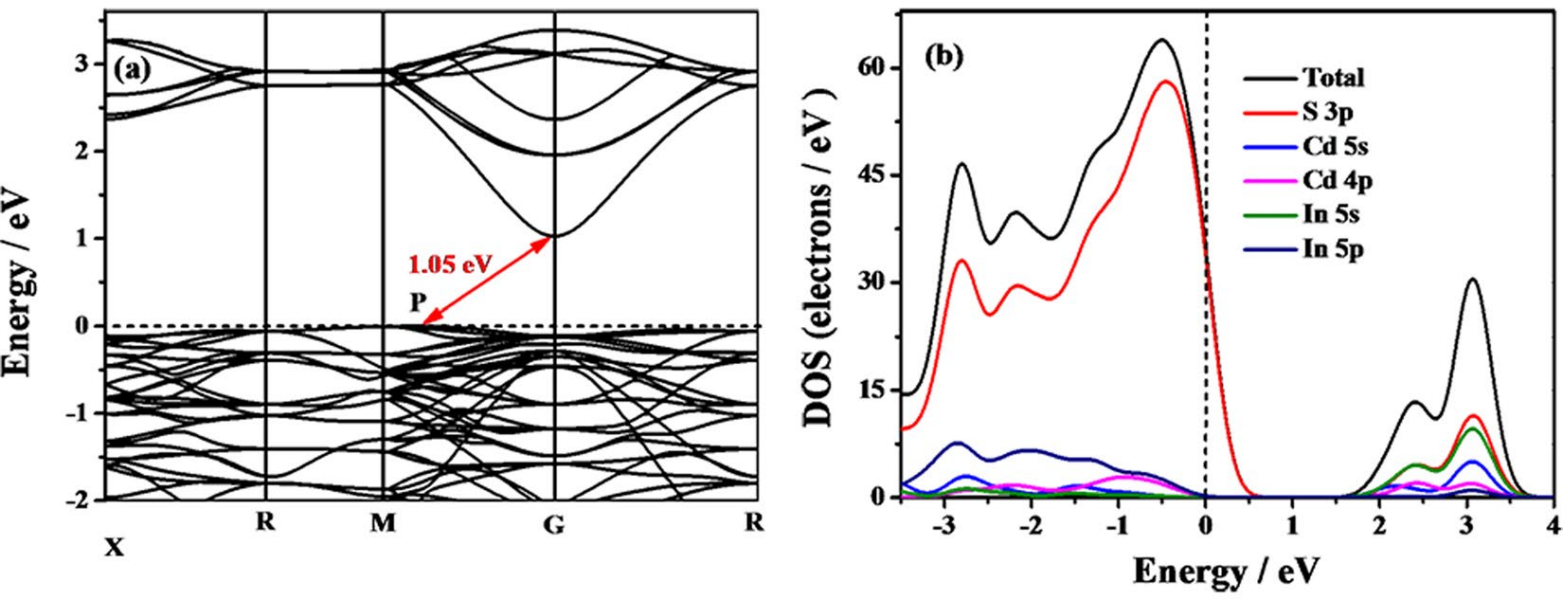
The band structure (**a**) and DOS (**b**) of CdIn_2_S_4_.

In order to clarify the electronic properties of CdIn_2_S_4_, the band structures and DOS of CdIn_2_S_4_ were calculated, as shown in Fig. 2. The Fermi level, shown by a dashed line, is set as zero. The conduction band minimum (CBM) is at G point, and the valence band maximum (VBM) is not at the high symmetry point but located in the direction of M → G (Fig. 2a), which indicates that CdIn_2_S_4_ is an indirect band gap semiconductor. With regard to the recombination of the photogenerated charge carriers, the indirect band gap semiconductors are more advantageous than the direct ones since the recombination of the photogenerated electrons and holes for indirect band gap needs the participation of phonon^30^. The calculated band gap for CdIn_2_S_4_ is 1.05 eV, which is much smaller than the experimental results because of limitation of DFT calculation. The DOS for CdIn_2_S_4_ indicates that the valence bands mainly consist of S states and a few In 5s, 5p and Cd 5s, 4p states, implying the existence of sp hybridizations among S 3p, In 5s, 5p and Cd 5s, 4p states. Meanwhile, the conduction bands are primarily composed of S 3p and In 5s states, mixed with a few Cd 5s, 4p states. This indicates that the sp hybridizations among S 3p, In 5s, 5p and Cd 5s, 4p states are existent in the valence and conduction band, which broadens the valence and conduction bands and promotes the transfer of the photogenerated carriers. These results indicate that CdIn_2_S_4_ can potentially serve as a high activity photocatalyst.

As is well known, the mobility ability of the photoexcited electrons and holes play an important role in the quantum efficiency of photocatalyst^31^. Moreover, the mobility of photogenerated carriers can be indirectly assessed by their effective masses. Generally, the mobility of the charge carrier is inversely proportional to their effective mass. To further analyze the mobility of photocatalytic activity of CdIn_2_S_4_, the effective masses of photogenerated charge carriers were calculated according to the following equation^32^:

1 $${m}^{\ast }=\pm {\hslash }^{{2}}/({{\rm{d}}}^{2}{E/\mathrm{dK}}^{{\rm{2}}})$$

Where *m* ^*^ is the effective mass of charge carriers; *k* is the wave vector; *ћ* is the reduced Planck constant; *E* is the band edge energy as a function. The effective masses of electrons around CBM are ca. 0.05 m_0_ and 0.04 m_0_ along the G → R and G → M directions, respectively. The effective masses of holes around CBM are ca. 0.96 m_0_ and 1.01 m_0_ along the P → G and P → M directions, respectively. In contrast to the reported values, the effective mass of photogenerated carriers in CdIn_2_S_4_ is smaller than that of other catalysts^33^. Due to small effective mass, it is clear that the electrons and holes can fast transfer to the surface of CdIn_2_S_4_. Moreover, the electron effective mass is much smaller than that of the hole, resulting in a great difference in the mobility between the electrons and holes. This large difference in mobility is beneficial to the separation of the carriers, inhibition of their recombination rate and enhancement of the photocatalytic activity.

### Characterization of CdIn_2_S_4_

#### XRD analysis

**Figure 3:**
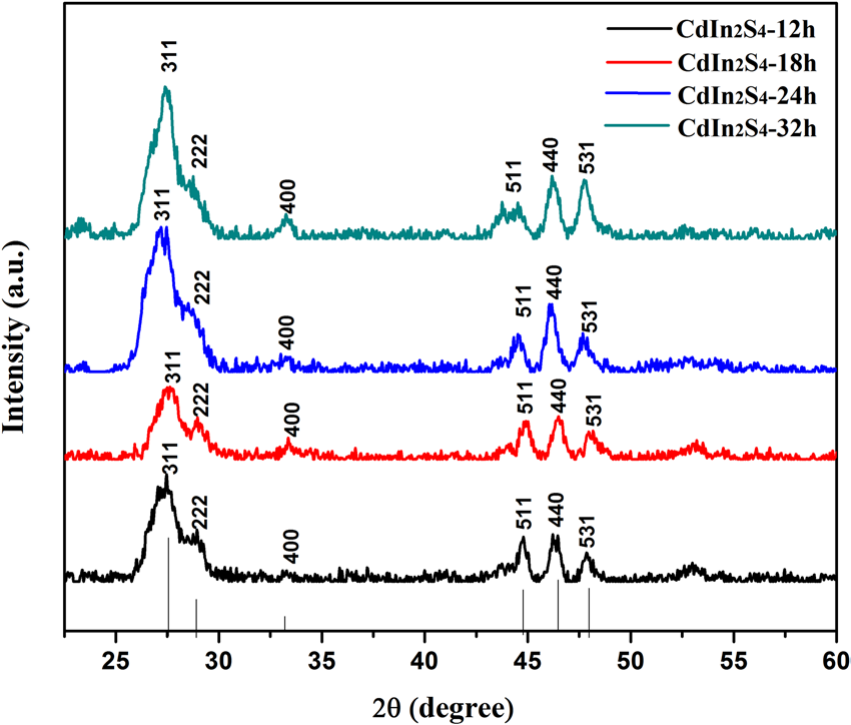
XRD patterns of CdIn_2_S_4_ photocatalysts prepared at different times.

The crystal phase composition and the crystallographic structure of the obtained products were determined by X-ray powder diffraction (XRD). Figure 3 shows the XRD patterns of CdIn_2_S_4_ photocatalysts with different preparation times for 12, 18, 24, 32 h, respectively. All the peaks in the patterns indicate that they belong to the cubic spinel structure and no characteristic diffraction peaks originated from CdS or In_2_S_3_ can be observed. The broad XRD peaks at 2 θ = 27.3° and 47.1° are the main characteristics of the cubic spinel phase of CdIn_2_S_4_ that are well indexed as (311) and (440) facets (JCPDS No. 27-0060), respectively. It is consistent with the previous reports^34^. Therefore, it can be concluded that a cubic-spinel phase CdIn_2_S_4_ photocatalyst is successfully prepared by a simple solvothermal method. Moreover, compared with the standard diffraction pattern of CdIn_2_S_4_, the broadness of the reflection is primarily due to the nanocrystalline nature of the samples. The reason may be that when ethylene glycol was used as a solvent, the growth of the sample was restricted on account of masking effect of ethylene glycol. Additionally, the (311) facet was chosen to calculate the crystallite size of the samples synthesized at different times. According to the Debyee-Scherrer equation, the average crystallite sizes of the samples are about 6.1 nm, and all the samples synthesized at the different times are almost similar. This can be confirmed by the experimental results, i.e. the photocatalytic activities of the photocatalysts synthesized at different times are almost similar. In the following experiments, reaction time for 12 h was selected to prepare catalyst.

#### UV-vis DRS analysis

**Figure 4:**
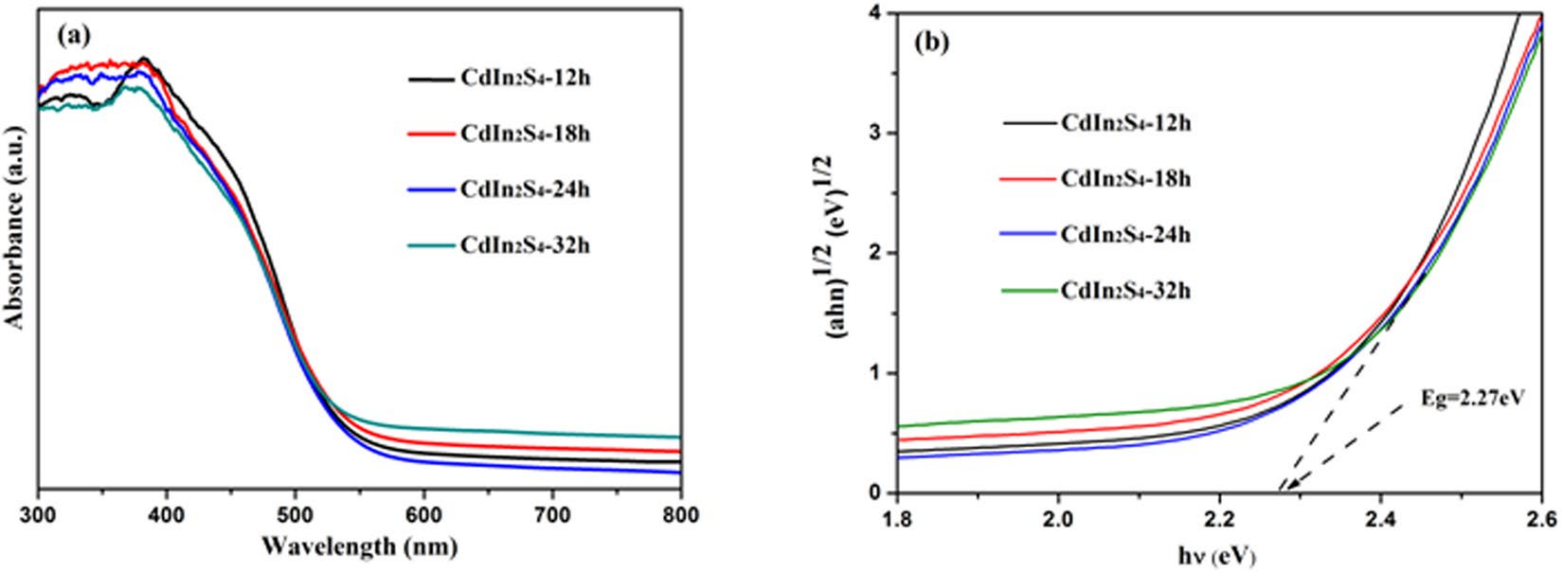
UV-vis diffuse reflectance spectra of CdIn_2_S_4_ synthesized at different times (**a**) and band gap energies of CdIn_2_S_4_ samples (**b**).

The optical properties of the samples, which are determined by UV-vis DRS, are displayed in Fig. 4a. It is quite clear that CdIn_2_S_4_ exhibits strong absorption in the visible light region from 400 to 550 nm and the absorption edges of CdIn_2_S_4_ synthesized at 12, 18, 24, 32 h display almost the same optical absorption. Moreover, because CdIn_2_S_4_ sample is an indirect transition semiconductor according to the afore mentioned demonstration, the band gap energy of CdIn_2_S_4_ particle is calculated by the following formula: αhv = A(hv − E_g_)^2^. Where α, A, h, and v are absorption coefficient, proportionality, Planck constant and light frequency, respectively^20^. As shown in Fig. 4b, the band gap of the CdIn_2_S_4_ is about 2.27 eV and CdIn_2_S_4_ semiconductor materials prepared at different preparation times have no obvious deviation. The band positions of CdIn_2_S_4_ photocatalysts also can be calculated by the following empirical formulas: E_CB_ = X − 0.5Eg − 4.5; E_VB_ = X + 0.5Eg − 4.5. Where X represents the average value of geometry of the Mulliken electronegativity of each constituent atom. After the calculation, it is found that the CB and VB positions are −0.819 and 1.351 eV, respectively.

#### SEM and TEM analysis

**Figure 5:**
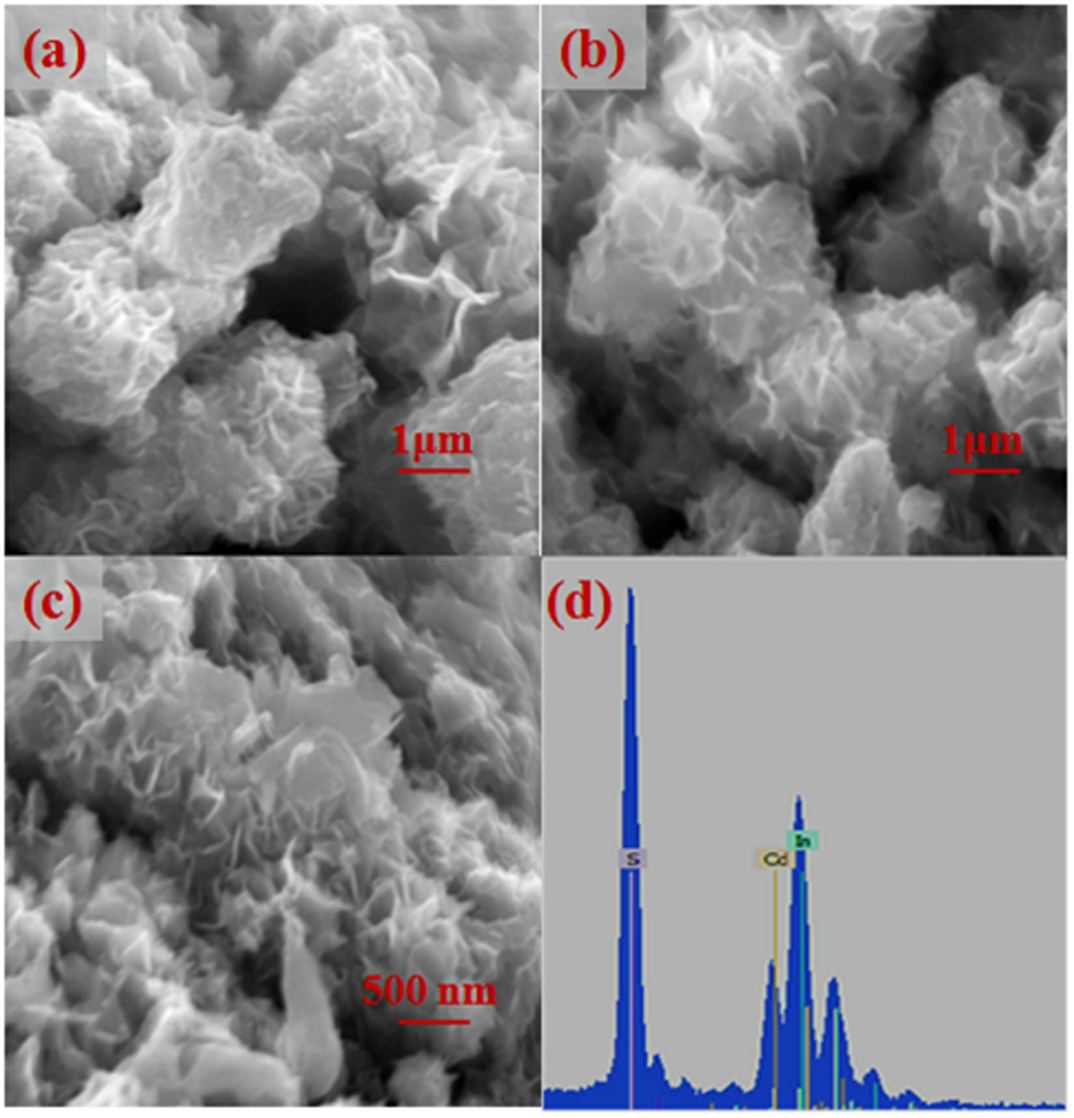
SEM images of CdIn_2_S_4_ sample synthesized at 160 °C for 12 h (**a–c**) and EDX pattern of CdIn_2_S_4_ sample (**d**).

Scanning electron microscopy (SEM) has been used to analyze the surface morphology of CdIn_2_S_4_ sample, as shown in Fig. 5(a,b). CdIn_2_S_4_ crystallites are self-organised into oblatoid assemblies or a coralloid-like morphology with a puffy appearance. Meanwhile, the high magnification image (Fig. 5c) clearly shows that CdIn_2_S_4_ crystallites are self-structured into coralloid-like morphology comprising numerous immature CdIn_2_S_4_ nano-petals. It is clear that CdIn_2_S_4_ sample shows significant aggregation with a diameter ranging from 2–4 μm and the gap between particles (nano-petals) was observed in the range of 200–600 nm. Additionally, the energy dispersive X-ray spectroscopy (EDX) demonstrates that the sample contains the signals of Cd, In, S elements, and the atomic ratio is 14.44: 28.39: 57.17, which conforms to the proportion of CdIn_2_S_4_ photocatalyst (Fig. 5d).

**Figure 6:**
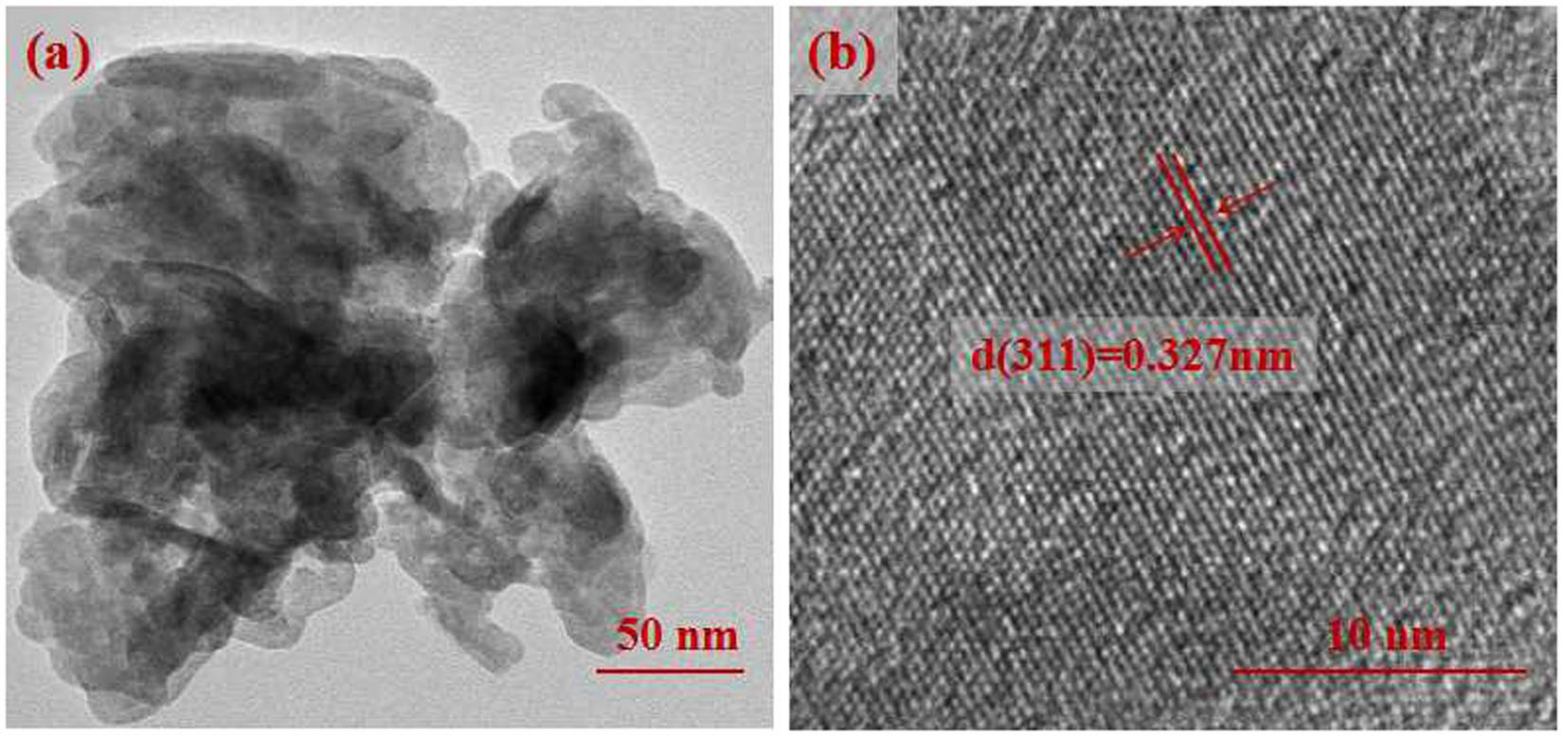
TEM (**a**) and HR-TEM images (**b**) of CdIn_2_S_4_ nano-petals synthesized at 160 °C for 12 h.

To further obtain the microscopic structure information, transmission electron microscopy (TEM) analysis of the CdIn_2_S_4_ sample was carried out, as displayed in Fig. 6. From Fig. 6a, it is obvious that the CdIn_2_S_4_ sample is consisted of small nanoparticles and has a thin sheet structure, which is in accordance with the SEM analysis. The image of high-resolution TEM (HRTEM) in Fig. 6b shows a distinct lattice fringe of CdIn_2_S_4_. The clear lattice spacing is measured to be 0.327 nm, which is consistent with the (311) crystal plane of XRD analysis.

#### XPS analysis

**Figure 7:**
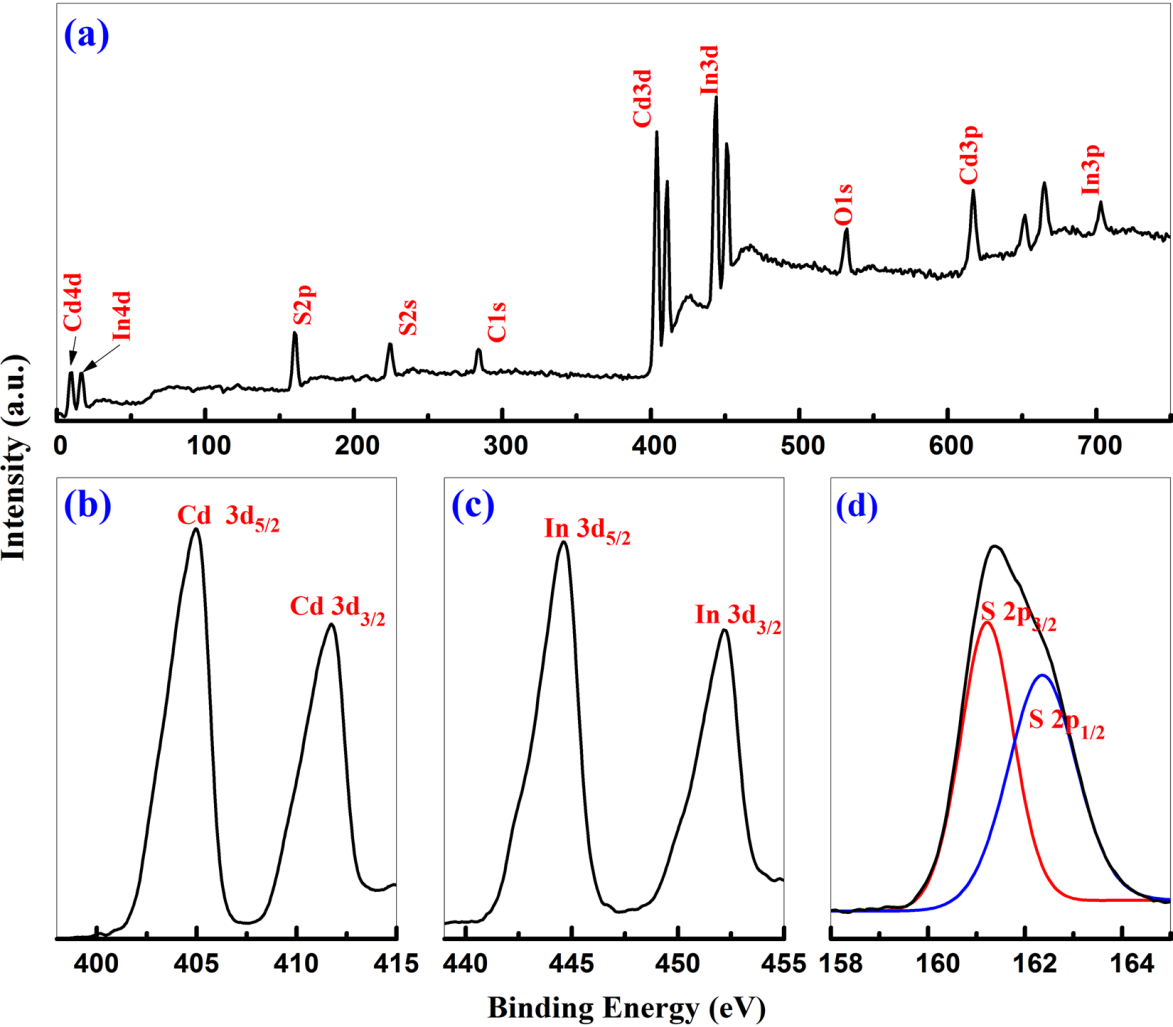
XPS spectra of CdIn_2_S_4_ photocatalyst synthesized at 160 °C for 12 h. Survey spectrum (**a**), and high-resolution spectra of Cd3d, In 3d and S2p, respectively (**b–d**).

The surface nature of the as-prepared CdIn_2_S_4_ sample was characterized by XPS measurements. From Fig. 7a, the survey spectrum of CdIn_2_S_4_ reveals that no peaks of other elements except Cd, In, S, C, O are observed. However, the oxygen peak at a binding energy of 533.2 eV and the carbon peak at a binding energy of 284.2 eV are inevitable. They come from the absorbed gaseous molecules and graphite conductive adhesive in all probability. High resolution core spectra for Cd 3d, In 3d, S 2p are shown in Fig. 7(b–d). From the figures, it is obvious that the peaks at the binding energy of 404.7 and 412.2 eV correspond to Cd 3d_5/2_ and Cd 3d_3/2_, the peaks at 444.6 and 452.3 eV are assigned to In 3d_5/2_ and In 3d_3/2_, and the peak at 161.3 and 162.5 eV could be attributed to S 2p_3/2_ and S 2p_1/2_, respectively^35^. The Cd, In, and S spin orbit separations are found to be 7.5, 7.7, and 1.2 eV, and the ration of the three peaks are about 2:3, 2:3 and 1:2. The above results indicate that Cd, In, and S are performed as Cd^2+^, In^3+^ and S^2−^ in CdIn_2_S_4_ photocatalyst. The peak areas of these high-resolution scans were measured and used to calculate the element ratio of the nanocrystals. The quantification of the peaks which gives the atomic ratio of CdIn_2_S_4_ photocatalyst in surface is in good agreement with the EDX analysis. Based on the results, it is suggested that the CdIn_2_S_4_ photocatalyst can be fabricated by a simple solvothermal method and the catalyst will have good applied prospect in photocatalytic selective transformation of organic compounds.

#### BET surface areas and pore size distributions analysis

**Figure 8:**
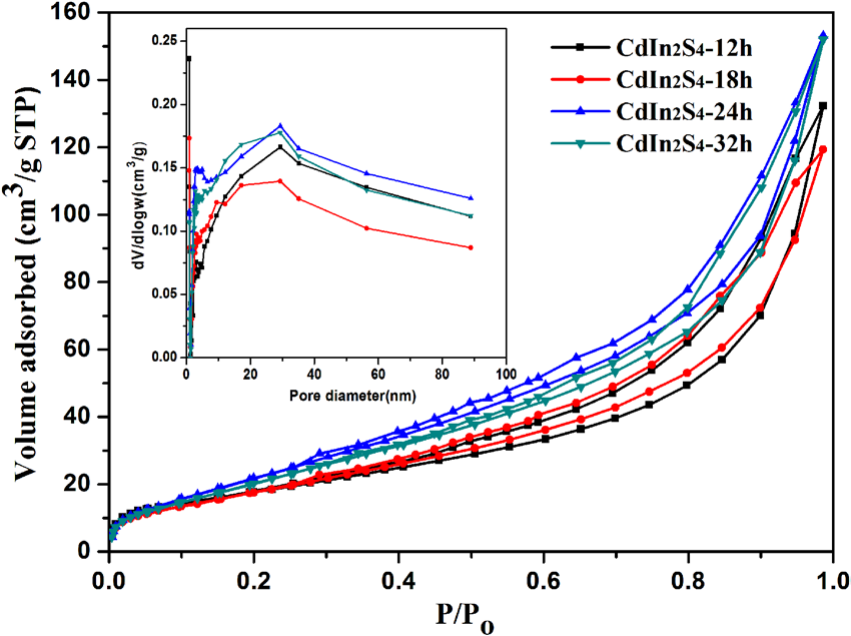
Nitrogen adsorption-desorption isotherms and corresponding pore-size distribution curves (inset) of the CdIn_2_S_4_ photocatalysts.

From Fig. 8, it can be seen that the nitrogen adsorption-desorption isotherm of CdIn_2_S_4_ photocatalyst exhibits type IV with a typical H3 hysteresis loop according to the IUPAC classification, implying that the samples have mesoporous structures^36^. It is known that the typical H3 loop is derived from aggregation of plate-like particles into slit-shaped pores. The result is consistent with that of the SEM and TEM analysis. Meanwhile, it is worth noting that the N_2_ adsorption-desorption isotherms of the samples prepared at different preparation times are similar. The BET surface areas of the samples are about 67.2, 73.0, 82.0, and 71.3 m^2^ g^−1^ with preparation times for 12, 18, 24, and 32 h, respectively. It can be seen that the samples have extremely high specific surface area, which would be beneficial for enhancing their photocatalytic activities. The corresponding pore-size distribution curves (inset in Fig. 8) of CdIn_2_S_4_ photocatalysts at different synthetic times exhibit a relatively wide pore-size distribution, which will provide efficient transport pathways for reactants and product molecules. In addition, the pore volumes of the samples prepared for 12, 18, 24 and 32 h are ca. 0.2047, 0.1846, 0.2268, and 0.2251 cm^3^ g^−1^, respectively.

Based on the above results, it is obvious that the CdIn_2_S_4_ photocatalysts prepared at different synthetic times have similar BET characterization data. Because of the afore mentioned excellent visible-light absorption and large BET surface area, it is believed that CdIn_2_S_4_ would be a promising photocatalyst.

### Evaluation of photocatalytic activities

**Figure 9:**
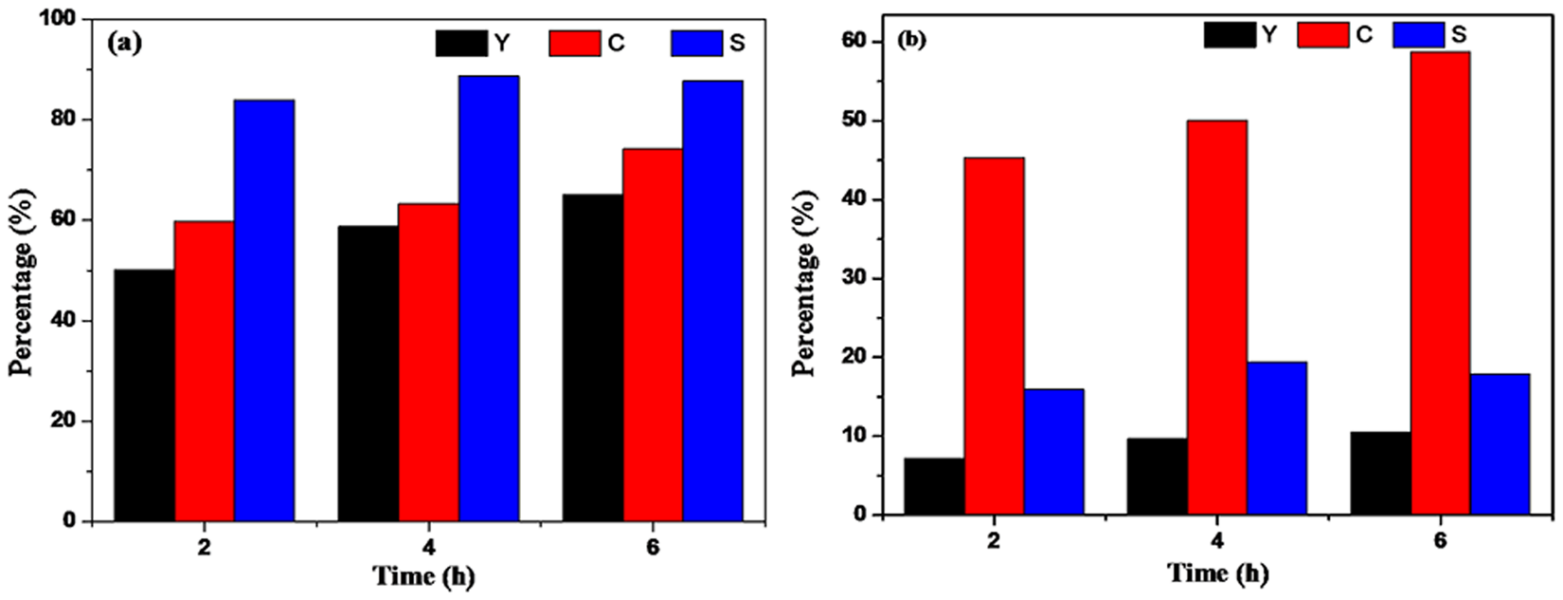
Typical time course of photocatalytic performance for selective oxidation of benzyl alcohol to benzaldehyde and reduction of nitrobenzene into aniline with visible light (λ> 420 nm) with N_2_ purge. benzyl alcohol (**a**), nitrobenzene (**b**) (In Figures, C, Y, and S mean Conversion, Yield, and Selectivity, respectively).

The photocatalytic activity of the as-obtained CdIn_2_S_4_ photocatalyst is initially studied by selective oxidation of benzyl alcohol to benzaldehyde and reduction of nitrobenzene into aniline with visible light irradiation (λ > 420 nm) for different times under N_2_ purge condition. We have verified that the CdIn_2_S_4_ photocatalysts synthesized at different times almost have similar photocatalytic activity (Fig. S1, Supplementary Information). For convenience, CdIn_2_S_4_ photocatalyst synthesized at 12 h was selected as a model sample for the following experiments. As shown in Fig. 9, it is clear that CdIn_2_S_4_ photocatalyst can realize the selective oxidation of benzyl alcohol to corresponding benzaldehyde and the reduction of nitrobenzene into aniline. For the selective oxidation of benzyl alcohol to the benzaldehyde, the result reveals that the conversion and yield increase gradually with the extension of the reaction time. With illumination time for 2 h, the conversion and yield are 59.7% and 50.1%, and with illumination time for 4 h, the conversion and yield are 66.3% and 58.8%. When the illumination time is 6 h, the conversion and yield are 74.2% and 65.3%, respectively. Meanwhile, it also can be seen that the selectivity increases with the extension of reaction time, which implies that the reaction is continuously carried out in a positive direction.

For the reduction of nitrobenezene into aniline^37^, the obtained results have roughly the same trend as the selective oxidation of benzyl alcohol. However, compared with the yield of benzaldehyde, the increase of aniline yield is relatively small. When the reaction time is 6 h, the conversion of nitrobenzene and yield of aniline are 57.4% and 10.5%, respectively, which are higher than the values obtained from 2 and 4 h. It further demonstrates that the reaction is continuously carried out in a positive direction. Notably, the conversion rate of nitrobenzene improves with the increase of reaction time, while the selectivity of the reaction shows no obvious increase when the reaction times are 4 h and 6 h, respectively. It indicates that the consumed nitrobenzene cannot be converted to aniline thoroughly with the extension of reaction time, probably because of another reaction co-existed in this system. Furthermore, a series of control experiments for 2 h were carried out (no catalyst but in the presence of visible light, or without visible light irradiation but in the presence of catalyst)^38^. As displayed in Fig. S2, no additional product was detected except benzyl alcohol and nitrobenzene under the same conditions, which confirms that the redox reactions are indeed driven by a photocatalytic process.

**Figure 10:**
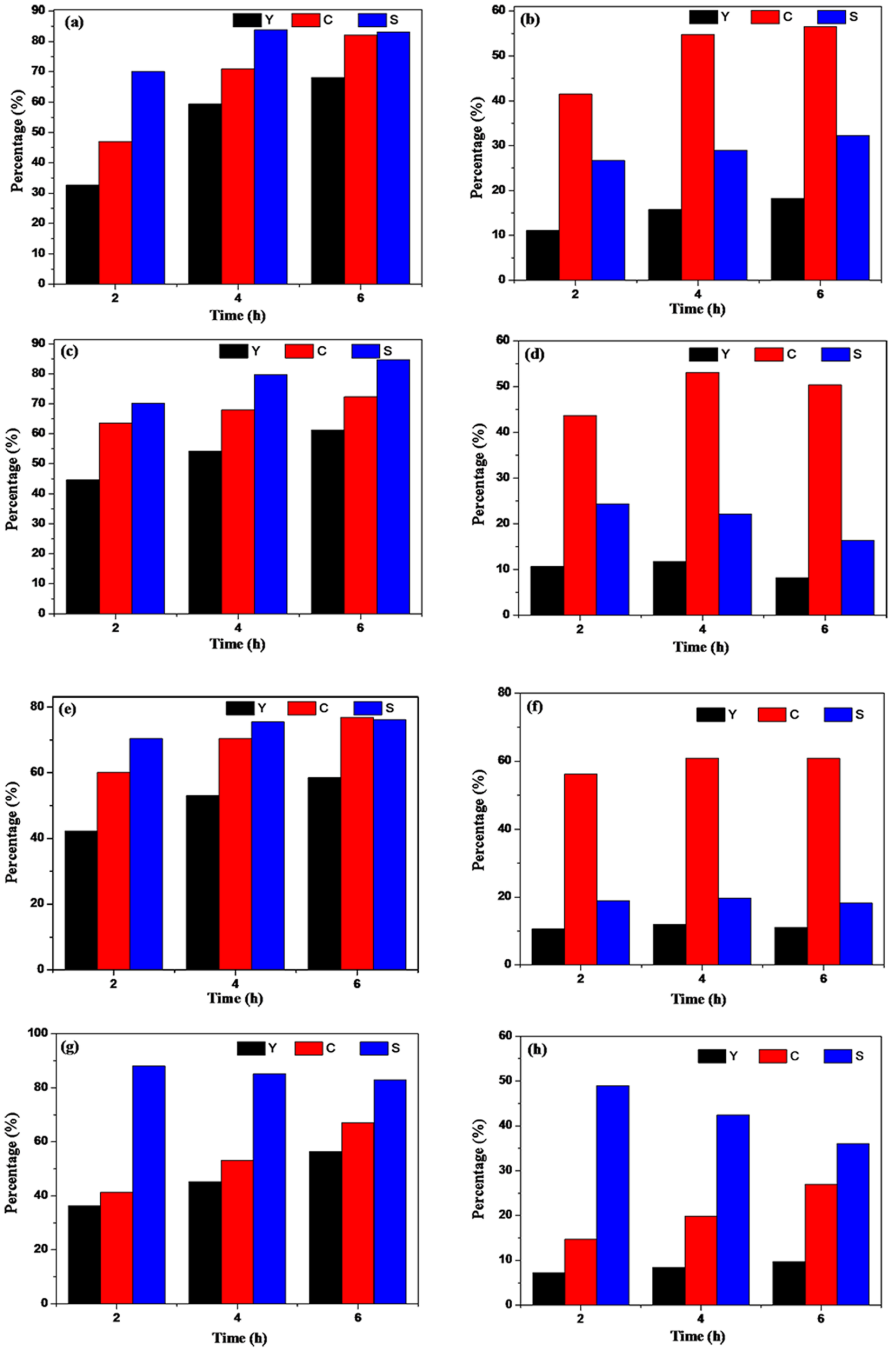
Photocatalytic performance of CdIn_2_S_4_ photocatalyst for selective oxidation of aromatic alcohols to aldehydes (**a, c, e** and **g**) and reduction of nitrobenzene into aniline (**b, d, f** and **h**) under visible light irradiation (λ > 420 nm) for different times in N_2_ purge condition. (**a**) p-methoxybenzylalcohol, (**b**) corresponding nitrobenzene; (**c**) p-chlorobenzylalcohol, (**d**) corresponding nitrobenzene; (**e**) p-fluorobenzyl alcohol, (**f**) corresponding nitrobenzene; (**g**) p-tolylmethanol, (**h**) corresponding nitrobenzene.

To learn more about the general applicability of CdIn_2_S_4_ photocatalyst, a range of aromatic alcohols with different substituent groups were chosen to evaluate the photocatalytic performance. It has been reported that the activity is directly proportional to the electronegativity of functional groups in our previous work^18^. It is known that the electronic effect of the functional groups follows the orders: -OCH_3_> -CH_3_ > -H >-Cl > -F. So the conversion, yield and selectivity of the reaction should be as follows: p-methoxybenzyl alcohol > p-tolylmethanol> benzyl alcohol > p-chlorobenzyl alcohol > p-fluorobenzyl alcohol. From Fig. 10, it can be seen that, for the selective oxidation of benzyl alcohol, the order of the photocatalytic activities follow the above rule apart from oxidation of p-tolylmethanol. And for the reduction of nitrobenzene, the activity is almost the same trend as the selective oxidation of a range of alcohols. Meanwhile, with the extension of reaction time, the conversions are on the rise, but the selectivities of aromatic alcohols and nitrobenzene are dwindling gradually in this coupled system. For example, as shown in Fig. 10g, in the coupled system toward the selective oxidation of p-tolylmethanol, as well as the reduction nitrobenzene, the conversion of p-tolylmethanol increases with the increase of the reaction time, while the selectivity reduces. For the reduction reaction of nitrobenzene into aniline, the selectivity of the reaction for 2, 4, and 6 h are 49.0%, 42.4%, and 36.0%, respectively. However, the corresponding conversion rates are 14.7%, 19.8%, and 27.0%, respectively. It is obvious that the trend of the reduction reaction is consistent with that of the oxidation reaction. However, this result runs counter to our normal understanding. Thus, we believe that there probably exists an interesting reaction in the coupled system. It is proposed that a Schiff base reaction may take place between p-tolualdehyde and aniline generated in the system. This research will be described in our subsequent work in detail.

In order to further verify the high photocatalytic activity of CdIn_2_S_4_ photocatalyst, the selective oxidation of benzyl alcohol to benzaldehyde in oxygen atmosphere was also selected as another probe reaction^39, 40^. As shown in Fig. S3 (Supplementary Information), under visible light illumination (λ > 420 nm) for 1 h, the conversion of benzyl alcohol and the yield of benzaldehyde are about 61% and 60%, respectively, so the selectivity of benzaldehyde reaches up to 98%. When the irradiation time is 2 h, the conversion, yield, and selectivity is increased to 80.2%, 80.0%, and 99%, respectively. With the extension of reaction time, the system reaches a dynamic balance finally. It suggests that CdIn_2_S_4_ photocatalyst has excellent catalytic efficiency and the optimum reaction time is 2 h. The results also show that higher oxygen pressure is not obviously favorable for the photocatalytic reaction. The optimal oxygen pressure is 0.1 Mpa (Fig. S4, Supplementary Information). Moreover, we have observed that superoxide radicals (O_2_^−^) and holes (h^+^) are the dominating active species in the process of the selective oxidation of benzyl alcohol to benzaldehyde in oxygen atmosphere (Fig. S5, Supplementary Information). CdIn_2_S_4_ photocatalyst is relatively stable and can be used repeatedly through cyclic experiments (Fig. S6, Supplementary Information).

In order to illustrate why CdIn_2_S_4_ has a higher photocatalytic activity, a series of control experiments between In_2_S_3_, CdS and CdIn_2_S_4_ samples (prepared in the same condition) were carried out. The results are listed in Fig. S7 (Supplementary Information). The results exhibit that CdIn_2_S_4_ photocatalyst has an outstanding photocatalytic activity for selective oxidation of aromatic alcohols to corresponding aldehydes. Thus, it is concluded that CdIn_2_S_4_ sample is an efficient and excellent photocatalyst.

#### Photocatalytic stability of CdIn_2_S_4_ photocatalyst

**Figure 11:**
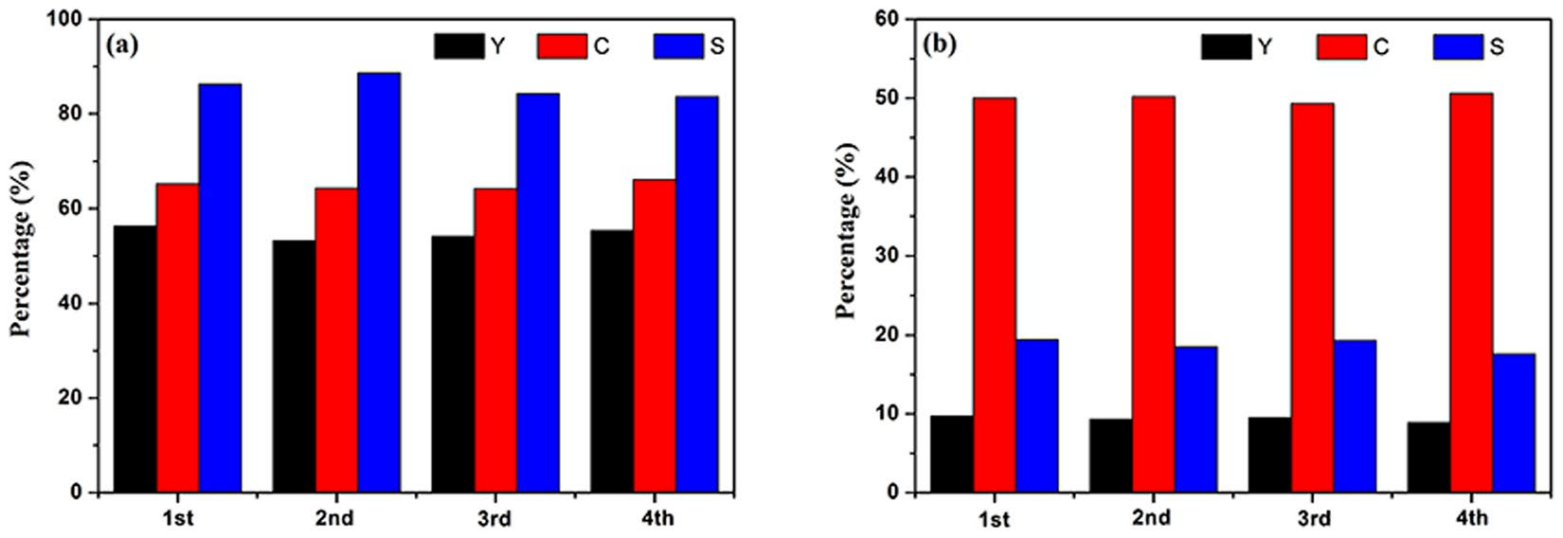
Cyclic experiments for selective oxidation of benzyl alcohol to benzaldehyde and reduction of nitrobenzene into aniline under visible light irradiation over CdIn_2_S_4_ photocatalyst in nitrogen condition for 4 h. (**a**) benzyl alcohol; (**b**) nitrobenzene.

**Figure 12:**
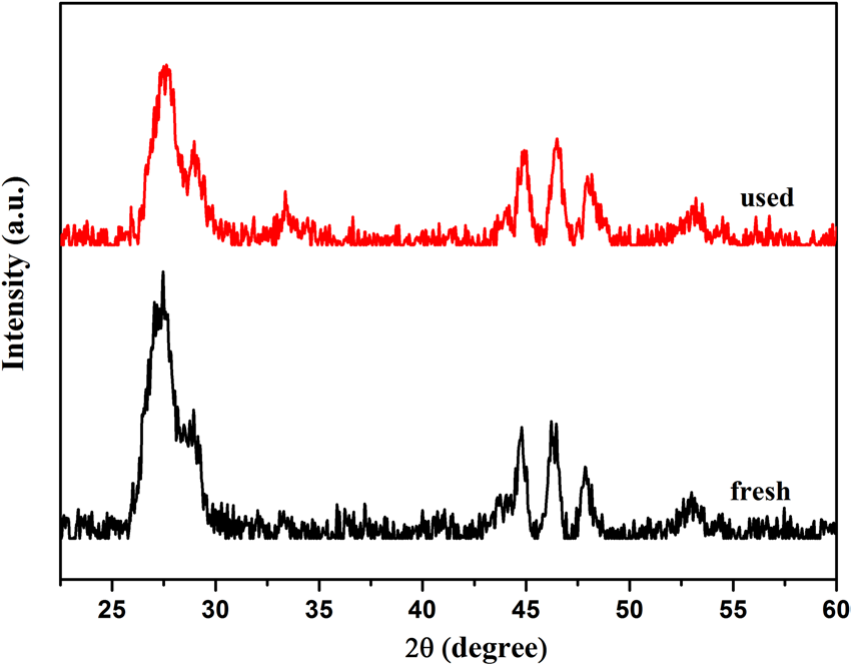
XRD patterns of CdIn_2_S_4_ photocatalyst before and after the photocatalytic reactions.

So as to evaluate the photocatalytic stability of CdIn_2_S_4_ photocatalyst for oxidation of aromatic alcohol to corresponding aldehyde and reduction of nitrobenzene to aniline, the cyclic experiments of CdIn_2_S_4_ photocatalyst were carried out under visible light irradiation (λ > 420 nm) for 4 h. After each experiment, the suspension was taken from the reaction kettle and centrifuged at 12000 rpm for 10 min to collect the catalyst. Then the catalyst was washed two times with deionized water and ethyl alcohol, respectively, followed by drying in air at 70 °C for 8 h. The result is summarized in Fig. 11. It indicates that the conversion of benzyl alcohol and nitrobenzene, as well as the yield of benzaldehyde and aniline do not have distinct change after cycle experiments for 4 times. To determine the nature of the sample, the fresh and used photocatalysts were investigated by XRD, as shown in Fig. 12. It is clear that the XRD patterns of the fresh and used samples also have no noticeable change. The results reveal that the CdIn_2_S_4_ is a highly active, stable, and reusable visible light-driven photocatalyst for selective oxidation of aromatic alcohol and reduction of nitrobenzene.

#### Photoelectrochemical properties

**Figure 13:**
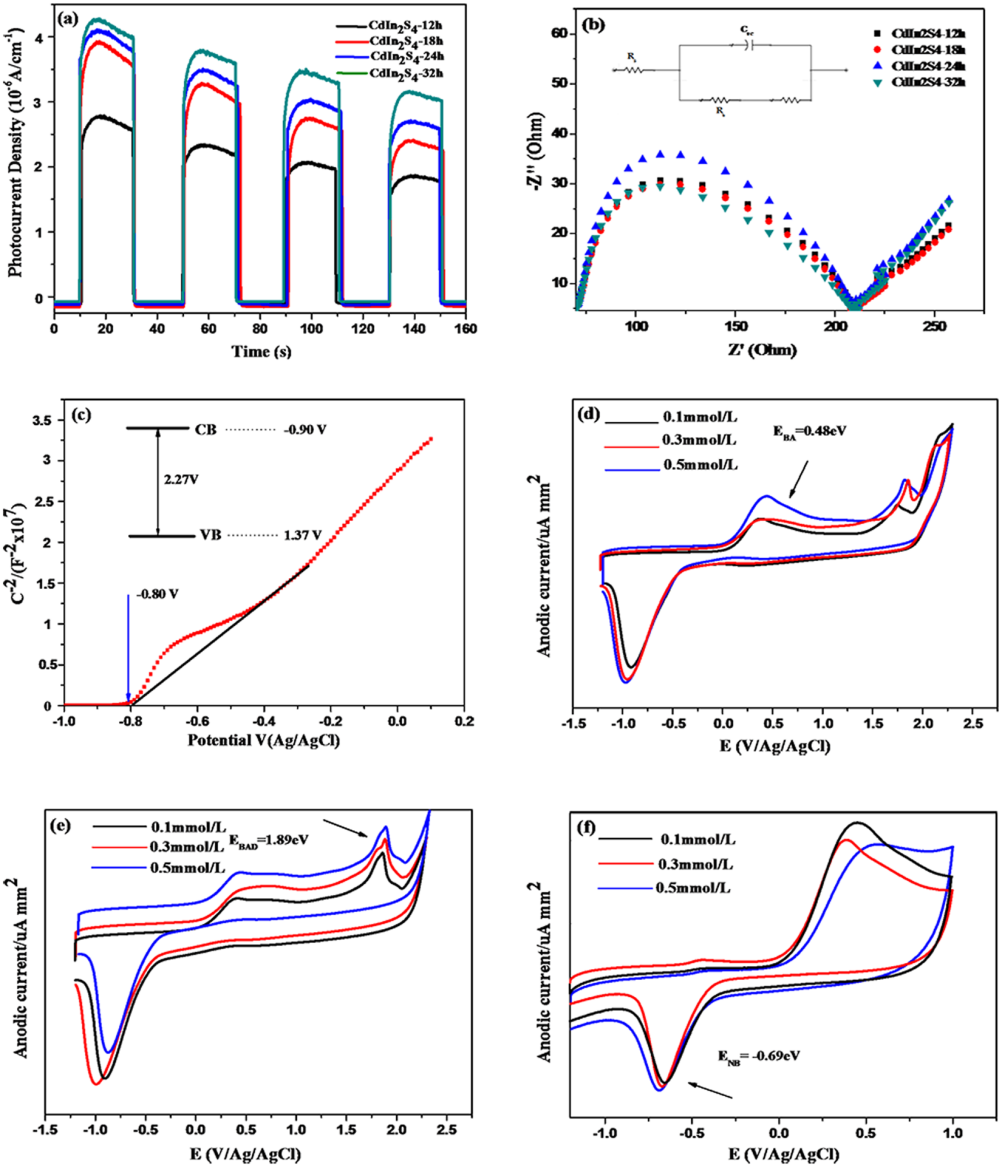
The photocurrent transient response of the samples in a 0.2 M Na_2_SO_4_ aqueous solution without bias versus Ag/AgCl electrode under visible light irradiation (**a**). EIS Nyquist plots of CdIn_2_S_4_ photocatalysts synthesized at different time in 0.1 M KCl solution containing 0.1 M K_3_[Fe(CN)_6_]/K_4_[Fe(CN)_6_] (**b**). (Inset: Electrical equivalent circuit used for fitting of impedance spectra, R_S_, C_SC_, R_b_ and W represent electrolyte resistance, space charge capacitance, bulk electrode resistance and Warburg impedance, respectively.) Mott-Schottky plots for the CdIn_2_S_4_ electrodes (**c**). The cyclic voltammogram of BA (**d**), BAD (**e**), and NB (**f**) with different concentrations (0.1, 0.2 and 0.5 mmol/L).

Photoelectrochemical experiments were performed to explore the origin of the photocatalytic performance of CdIn_2_S_4_ photocatalysts synthesized at different times for 12, 18, 24, and 32 h, respectively^41^. Figure 13a displays the photocurrent transient response under intermittent visible light irradiation. It can be seen that the photocurrent increases gradually with the increase of preparation time, and the photocurrent rapidly decreases to zero as long as the light is switched off. The CdIn_2_S_4_ prepared for 32 h has the highest photocurrent transient response in the all examined samples, suggesting the remarkably efficient separation of charge carriers upon visible light irradiation. However, the result exhibits that the CdIn_2_S_4_ samples prepared at different synthetic times have similar photocatalytic activities. Based on the above results, it is concluded that the lifetime of the photogenerated charge carriers is one of the most key factors for the photocatalytic performance, but other factors such as specific surface and crystalline also play an important role in determining the photocatalytic performance. They are a synergetic action. Although the photocurrent density has regular difference, it does not violate the conclusion that the CdIn_2_S_4_ synthesized at different times have similar photocatalytic activities.

To continue to research the origins of the excellent photocatalytic performance and the reason why the photocatalyst synthesized at different synthetic times have the similar activities, Electrochemical impedance spectroscopy (EIS) experiments in the dark has been carried out^20^. EIS Nyquist plots of the CdIn_2_S_4_ electrode materials cycled in 0.1 M KCl aqueous solution containing 0.1 M K_3_[Fe(CN)_6_]−K_4_[Fe(CN)_6_] (1:1) are shown in Fig. 13b. From the EIS of the CdIn_2_S_4_ photocatalysts synthesized at different reaction times for 12, 18, 24, and 32 h, it can be seen that the arc radius are similar and resistance value is quite small. It is known that the smaller resistance value of the EIS Nyquist plots corresponds to the more effective separation of the photoinduced electron-hole and the faster interfacial charge transfer. This result can explain why CdIn_2_S_4_ photocatalysts synthesized at 160 °C in ethylene glycol have so high photocatalytic activity. The similar arc radius of CdIn_2_S_4_ photocatalysts synthesized at different times is just in agreement with the results of photocatalytic activities.

We also carried out the electrochemical analysis of the Mott-Schottky plots in the dark. As shown in Fig. 13c, the plots with the positive slope are observed, which is consistent with the typical feature of n-type semiconductor. The flat band potential of CdIn_2_S_4_ photocatalysts synthesized at 160 °C for 12 h, as calculated from the X intercepts of the linear region, is found to be approximately −0.80 eV vs. Ag/AgCl. It is known that the bottom of the conduction band is more negative by ∼−0.1 V than the flat band potential for many n-type semiconductors, and the converted potential of the NHE versus Ag/AgCl is 0.2 eV. Therefore, the CB and VB positions are −0.70 and 1.57 eV vs. NHE, respectively. The experimental test values obtained here are close to the result of the theoretical calculation of DRS analysis.

In order to examined the feasibility of the photocatalytic redox reaction, the redox potentials of benzyl alcohol (BA), benzaldehyde (BAD), and nitrobenzene (NB) with different concentrations (0.1, 0.2, 0.5 mmol/L) were evaluated using cyclic voltammetry, respectively. As shown in Fig. 13d, the cyclic voltammogram of BA has two oxidation potentials. The first oxidative potential is BA to BAD at *ca.* +0.48 eV vs. Ag/AgCl, and the second one belongs to BAD/corresponding acid. The oxidative potential is *ca.* +1.89 eV vs. Ag/AgCl, which is shown in Fig. 13e. In addition, the reductive potential of NB is −0.69 eV vs. Ag/AgCl (displayed in Fig. 13f). It is known that the conduction band of CdIn_2_S_4_ photocatalyst by Mott-Schottky experiments is −0.90 eV vs. Ag/AgCl, which is more negative than reductive potential of NB. The valence band is 1.37 eV (vs. Ag/AgCl) and is more positive than oxidative potential of BA to BAD, but is inadequate to oxidize BAD into benzoic acid. Therefore, the selective oxidation BA to BAD and reduction of NB to aniline can be achieved using CdIn_2_S_4_ photocatalyst in a coupled system under visible light illumination. These results are supported by the theory calculation and are also consistent with the experiment results.

### Reaction mechanism

**Figure 14:**
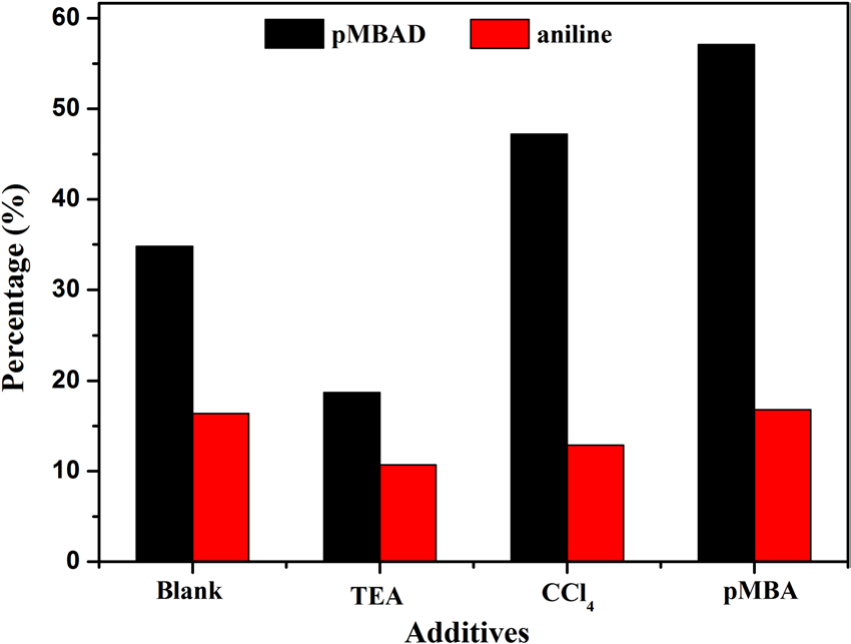
The yield of p-methoxybenzaldehyde (p-MBAD) and aniline over CdIn_2_S_4_ by adding different additives under visible light for 4 h.

In nitrogen condition, h^+^ and e^−^ are the major reactive species for selective redox transformation of organics^42, 43^. In order to investigate the specific reaction mechanism of free radicals in the reaction process, a series of control experiments were carried out. In this study, triethanolamine (TEA) and tetracholromethane (CCl_4_) were used as scavengers for trapping photogenerated holes and electrons, respectively. As shown in Fig. 14, when TEA was added into the reaction system, the yield of p-methoxybenzaldehyde (pMBAD) decreased obviously. Compared with the blank experiment, it is known that the photogenerated holes are decisive factors for the selective oxidation reaction. As a rule, when the photogenerated holes are scavenged in the reaction system, it must be favorable for the reduction reaction. Because of the decrease of the photogenerated holes, the combination of photogenerated carriers is inhibited and the corresponding remaining photoexcited electrons contribute to reducing nitrobenzene (NB)^44^. However, the addition of hole scavenger (TEA) did not improve the yield of aniline. Furthermore, when pMBA was not added into the reaction system, no conversion of NB was observed. It suggests that the oxidation reaction was on the premise of reduction of NB. This may be caused by the fact that protons (H^+^) are produced in the oxidation of pMBA and they are necessary for reduction of NB into aniline. H^+^ and photogenerated electron dominate the fate of reduction reaction. This conclusion can also be confirmed the following experiment. When more pMBA is added into the reaction system, the yield of pMBAD and aniline increased obviously, respectively. Thus, the selective redox organic transformations are synergetic and coadjutant in the coupled system^26^.

When CCl_4_ was added into the reaction system, the yield of aniline decreased significantly, implying that photogenerated electrons are the major active species in the reduction reaction. However, as shown in the Fig. 14, the yield of p-methoxybenzaldehyde (pMBAD) increased, suggesting that the trapping of photogenerated electrons is favorable for the selective oxidation of pMBA into pMBAD. This may be that the quenching of the electrons contributes to effectively inhibit the combination of the photogenerated carriers and then separate the photogenerated electron-hole pairs efficiently.

**Figure 15:**
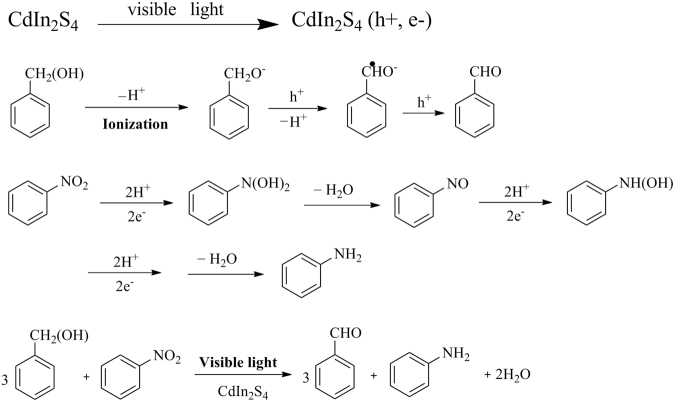
Proposed mechanism for the selective oxidation of benzyl alcohol to benzaldehyde and reduction of nitrobenzene into aniline in a coupled system under visible light.

According to the above experimental conclusions and our previous reports^45^, a possible mechanism for the photocatalytic selective oxidation of aromatic alcohols and reduction of nitrobenzene over CdIn_2_S_4_ photocatalyst under visible light was proposed in Fig. 15.

Upon visible light illumination, CdIn_2_S_4_ can be excited and produces a number of photogenerated electrons-holes pairs^46, 47^. Herein, the N_2_ environment provides an anaerobic atmosphere for the reaction. So, the nitrobenzene has no opportunity to carry out the oxidation reaction. Firstly, benzyl alcohol is deprotonated to produce alkoxide anions. Then, alkoxide anions react with holes of the valence band and sequentially deprotonate to form carbon radicals. The product is reacted with the holes to form benzaldehyde. Subsequently, the two generated protons and photogenerated electrons react with nitrobenzene. H_2_O molecular is lost and an intermediate product (nitrosobenzene) is generated. As the reaction progresses, the nitrosobenzene gradually turns into the final product (aniline). It is obvious that the photogenerated electrons and holes play the crucial roles in the redox coupling process. As shown in Fig. 15, completing reduction of one mol nitrobenzene needs six mol electrons. The six mol electrons are accompanied with six moles holes. Therefore, according to theoretical analysis, the amount of benzaldehyde should be three times of aniline. However, based on the activity data of selective redox reaction, the generated amount of aromatic aldehyde and aniline are not consistent with the above theoretical value. Therefore, it is considered that there should be a Schiff base reaction between aromatic aldehyde and aniline. Certainly, we will discuss the work in detail in the future.

## Conclusions

In summary, a cubic-spinel phase CdIn_2_S_4_ semiconductor with coralloid-like morphology was successfully synthesized by a simple solvothermal method. The photocatalyst exhibits excellent photocatalytic activity and high chemoselectivity toward the selective oxidation of aromatic alcohols to aromatic aldehydes and reduction of nitrobenzene into aniline in nitrogen atmosphere. And it also exhibits an outstanding photocatalytic activity for selective oxidation of aromatic alcohol to aromatic aldehydes under oxygen condition. The superior photocatalytic performance of CdIn_2_S_4_ is mainly attributed to its unique structure assembly of specific morphology with high surface area, as well as appropriate conduction band and valence band positions with efficient separation of the photogenerated charge carriers under visible light irradiation. So far, it is the first time to utilize CdIn_2_S_4_ sample as a visible light-driven photocatalyst for the selective oxidation and reduction of organics. The reasons why CdIn_2_S_4_ photocatalyst has a high photocatalytic activity are investigated. We hope that this work would open the doorway for the exploration of CdIn_2_S_4_ semiconductor and make ongoing efforts to synthesize or discover other new-style visible-light- driven photocatalysts for selective transformations of organics.

### Chemicals and Methods

#### Chemicals

Indium chloride tetrahydrate (InCl_3_^.^4H_2_O), cadmium nitrate tetrahydrate [Cd(NO_3_)_2_^.^4H_2_O], thiacetamide (TAA), ethylene glycol, as well as other reagents used in the experiments were purchased from Shanghai Chemical Reagent Co. Ltd of China. All of the reagents were analytical grade without further purification. Deionized water was used throughout this study.

#### Preparation of photocatalysts

In a typical process, the precursor solutions of Cd(NO_3_)_2_^.^4H_2_O, InCl_3_^.^4H_2_O, and thioacetamide (TAA) were prepared in ethylene glycol and mixed in the molar ratio of 1:2:4 followed by stirring for 40 min using magnetic stirrer. The obtained homogeneous phase solution was transferred into a100 mL Teflon-lined stainless-steel autoclave. Then the autoclave was maintained at 160 °C for 12 h, and naturally cooled to room temperature. The obtained product was collected by centrifugation, washed with deionized water and absolute ethanol 2 times to remove the residual organic solvents and inorganic salts, and then dried in air at 70 °C for 8 h.

#### Characterization

The powder X-ray diffraction (XRD) patterns of the samples were collected on a Bruker D8 Advance X-Ray diffractometer with Cu-K_α_ radiation and a scanning speed of 3°/min at room temperature. The crystallite size was calculated by X-ray line broadening analysis using the Scherrer equation. The accelerating voltage and the applied current were 40 kV and 40 mA, respectively. UV-vis diffuse reflectance spectra (DRS) of the obtained samples were carried out using a UV-3600 (SHIMA-DZU, Japan) UV-vis-NIR spectrophotometer equipped with an integrating sphere attachment. BaSO_4_ was used as a reflectance standard. The surface characteristics and microcrystalline structure were measured using ZEISS-EVO18 scanning electron microscope with 30 kV scanning voltages. Transmission electron microscope (TEM) and high-resolution transmission electron microscope (HR-TEM) images were performed with a JEM-2100 electron microscope with an accelerating voltage of 200 kV. X-ray spectroscopy (XPS) measurement was carried out on a Thermo Scientific ESCA Lab250 spectrometer, which consists of monochromatic Al Kα as the X-ray source, a hemispheric analyzer, and a sample stage with multiaxial adjustability to obtain the surface composition of the sample. The specific surface area and pore size distribution of the samples were determined by a Micromeritics ASAP 2020. All of electrochemical experiments were performed with a CHI-660E electrochemical workstation (CHI Instruments, USA). The photocurrent was carried out in a conventional three-electrode cell using a Pt plate and a Ag/AgCl electrode as the counter electrode and reference electrode, respectively. The catalyst powder deposited on the fluoride tin oxide (FTO) substrate was employed as the working electrode, and a quartz cell filled with 100 mL 0.2 M Na_2_SO_4_ electrolyte was used as the reaction system. A 300 W Xenon lamp (CEL-HXF300E7, Beijing China Education Au-light Co., Ltd) system was applied as the excitation light source equipped with a UV cutoff filter (λ < 420 nm), which was the same light source for the photocatalytic tests. Electrochemical impedance spectroscopy (EIS) measurements were recorded using a conventional three-electrode configuration. The electrolyte system consists of 0.1 M KCl and 0.1 M K_3_[Fe(CN)_6_]/K_4_[Fe(CN)_6_] solution. Mott-Schottky (M-S) experiments of the resulted samples were analyzed at the potential range −1.0 to 0.1 V with the amplitude of 0.01 at a constant frequency of 1000 Hz. The electrolyte system is composed of 0.2 M Na_2_SO_4_ solution. The Cyclic Voltammetry (CV) was measured by sweeping at 0.05 V/S in CH_3_CN/0.1 mol/L LiClO_4_/(0.1, 0.2 and 0.5 mmol/L) X (X is BA, BAD, or NB) solution. A glass carbon disk, a platinum wire and an Ag/AgCl electrode were used as the working electrode, counter electrode, and reference electrode, respectively.

#### Evaluation of photocatalytic activity

The photocatalytic activities of the photocatalysts for selective oxidation of aromatic alcohols to corresponding aromatic aldehydes and reduction of nitrobenzene (NB) into aniline were carried out in a 100 mL Teflon-lined stainless steel autoclave under visible light irradiation^26^. The initial concentrations of aromatic alcohol and NB were 2.55 × 10^−2^ and 8.5 × 10^−3^ mol/L, respectively. 15 mL reaction solution (benzotrifluorideas solvent) containing reactants and 80 mg photocatalyst were put into 100 mL reaction apparatus. The illumination window on the top of the reactor is made of high strength quartz glass. Before the illumination, nitrogen was passed through the solution for 20 min to remove the dissolved oxygen and filled with pure nitrogen (0.1 MPa). The suspension was stirred for 30 min to establish an adsorption-desorption equilibrium. A 300 W Xenon lamp with a UV-CUT filter to cut off light of wavelength below 420 nm was used as an irradiation source. Because of the continuous cooling with the refrigeration circulating water, the temperature of the reaction solution was maintained at approximately 60 °C. After the illumination for different times, the reaction solution was collected from the reactor and centrifuged at 12000 rmp for 10 min to remove the catalyst completely. The obtained reaction solutions were analyzed by gas chromatograph (Shimadzu GC-2014C, Japan) equipped with an SE-30 capillary column.

The main product of the selective oxidation of aromatic alcohol is aromatic aldehyde, and the reduction product of nitrobenzene (NB) is aniline. Conversion of alcohol, yield of aldehyde and selectivity for aldehyde were defined as follows^27^:

2 $${\rm{Conversion}}( \% )=[({{C}}_{{\rm{0}}}\mbox{--}{{C}}_{{\rm{alcohol}}})/{{C}}_{{\rm{0}}}]\times \mathrm{100} \% $$

3 $${\rm{Yield}}( \% )={{C}}_{{\rm{aldehyde}}}/{{C}}_{{\rm{0}}}\times \mathrm{100} \% $$

4 $${\rm{Selectivity}}( \% )=[{{C}}_{{\rm{aldehyde}}}/({{C}}_{{\rm{0}}}\mbox{--}{{C}}_{{\rm{alcohol}}})]\times \mathrm{100} \% $$

Where C_0_ is the whole amount of alcohols in the solution before illumination; C_alcohol_ is the amount of alcohols in the solution after illumination at a certain time; C_aldehyde_ is the amount of aldehydes in the solution after illumination at a certain time.

The reduction of NB was calculated as follows:

5 $${\rm{Conversion}}( \% )=[({{C}}_{{\rm{0}}}\mbox{--}{{C}}_{{\rm{NB}}})\,/{{C}}_{{\rm{0}}}]\times \mathrm{100} \% $$

6 $$\,{\rm{Yield}}( \% )={{C}}_{{\rm{aniline}}}/{{C}}_{{\rm{0}}}\times \mathrm{100} \% $$

7 $${\rm{Selectivity}}( \% )=[{{C}}_{{\rm{aniline}}}/({{C}}_{{\rm{0}}}\mbox{--}{{C}}_{{\rm{NB}}})]\times \mathrm{100} \% $$

Where C_0_ is the whole amount of NB in the solution before illumination; C_NB_ is the amount of NB in the solution after illumination at a certain time; C_aniline_ is the amount of aniline in the solution after illumination at a certain time.

#### Computational details

All DFT calculations for CdIn_2_S_4_ were performed by CASTEP code based on the plane-wave pseudopotential method^28^. The Perdew-Burke-Ernzerh (PBE) function form of generalized gradient approximation (GGA) was used as the exchange-correlation function^29^. The ultrasoft pseudopotential was adopted to describe the interaction between ionic core and valence electrons. The lattice parameters and atomic coordinates were relaxed using the cutoff of 400 eV and Monkhorst-pack grids of 3 × 3 × 3 k-points. The related parameters of convergence tolerance for geometry optimization calculations were a total energy of 5.0 × 10^−6^ eV/atom, a maximum force of 0.01 eV/Å, a maximum stress of 0.02 GPa and a maximum atomic displacement of 5.0 × 10^−4^ Å. The static self-consistent field calculation was terminated until the tolerance was less than 1 × 10^−6^ eV/atom. The valence electrons were taken into account for CdIn_2_S_4_ corresponding to Cd: 4p^6^4d^10^5s^2^, In: 4d^10^5s^2^5p^1^ and S: 3s^2^3p^4^, while the remained electrons were kept frozen as core states. The bigger cutoff energy and k-points were used to ensure the accuracy of calculation. The results indicated almost no change in the energy and geometry structure.

## Electronic supplementary material

Supplementary_Information
