# Supplementary material for: Solvothermal synthesis of CdIn2S4 photocatalyst for selective photosynthesis of organic aromatic compounds under visible light
Source: Sci Rep. 2017 Feb 9;7:27. doi: 10.1038/s41598-017-00055-5 (PMC5428363; doi:10.1038/s41598-017-00055-5)
Supplement: Supplementary file 1 — Supplementary_Information [file 41598_2017_55_MOESM1_ESM.pdf]

Supplementary Information for

Solvothermal synthesis of $CdIn_2S_4$ photocatalyst for selective photosynthesis of organic aromatic compounds under visible light

Cancan Ling[a,b], Xiangju Ye[b*], Jinghu Zhang[a,b], Jinfeng Zhang[a], Sujuan Zhang[a], Sugang Meng[a], Xianliang Fu[a] & Shifu Chen[a,b*]

[a] Department of Chemistry, Huaibei Normal University, Anhui Huaibei, 235000, People's Republic of China.

[b] Department of Chemistry, University of Science and Technology of Anhui, Anhui Fengyang, 233100, People's Republic of China.

* Corresponding author, Tel: +86-561-3806611, Fax: +86-561-3806611. E-mail: chshifu@chnu.edu.cn

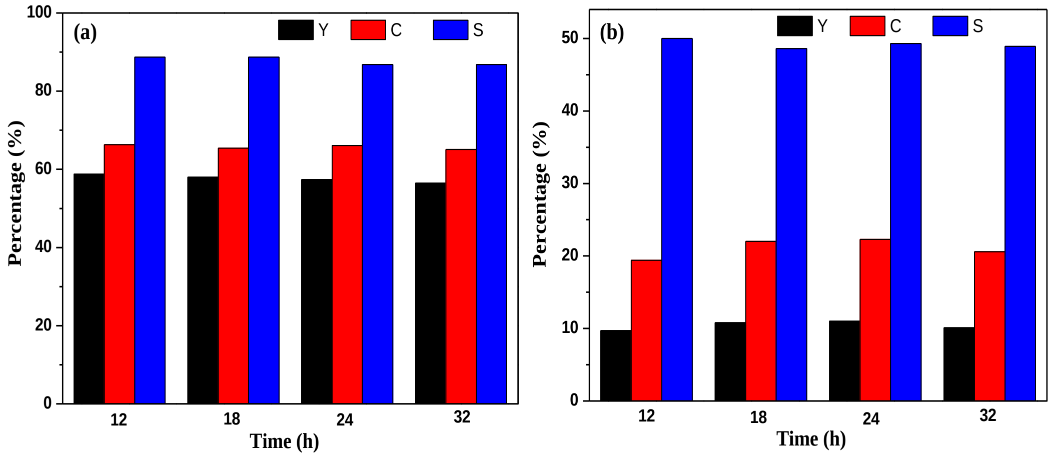

Fig. 1. Photocatalytic performance of the $CdIn_2S_4$ photocatalysts synthesized at different times for selective oxidation of benzyl alcohol to benzaldehyde and reduction of nitrobenzene into aniline under visible light irradiation (λ>420 nm) for 4 h in N_2 purge condition. (a) benzyl alcohol, (b) nitrobenzene.(In Figures, C, Y, and S represent Conversion, Yield, and Selectivity, respectively).

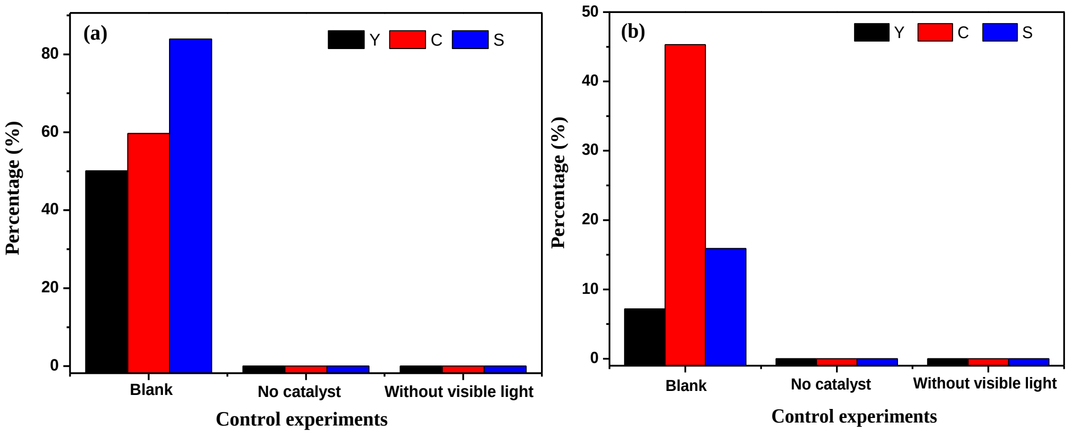

Fig. 2. Control experiments for selective oxidation of benzyl alcohol to benzaldehyde and reduction of nitrobenzene into aniline with visible light (λ>420 nm) with N_2 purge for 2 h. benzyl alcohol (a), nitrobenzene (b).

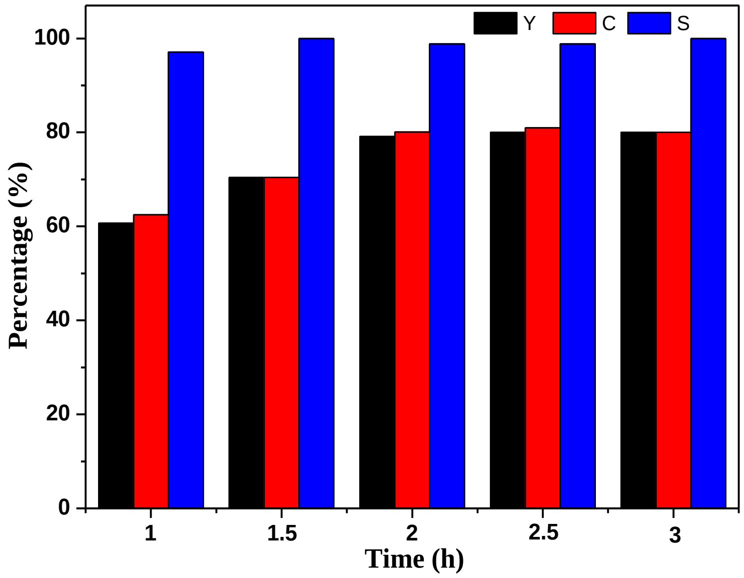

Fig. 3. Selective oxidation of benzyl alcohol to benzaldehyde under visible light irradiation (λ>420 nm) for different times under O_2 purge condition.

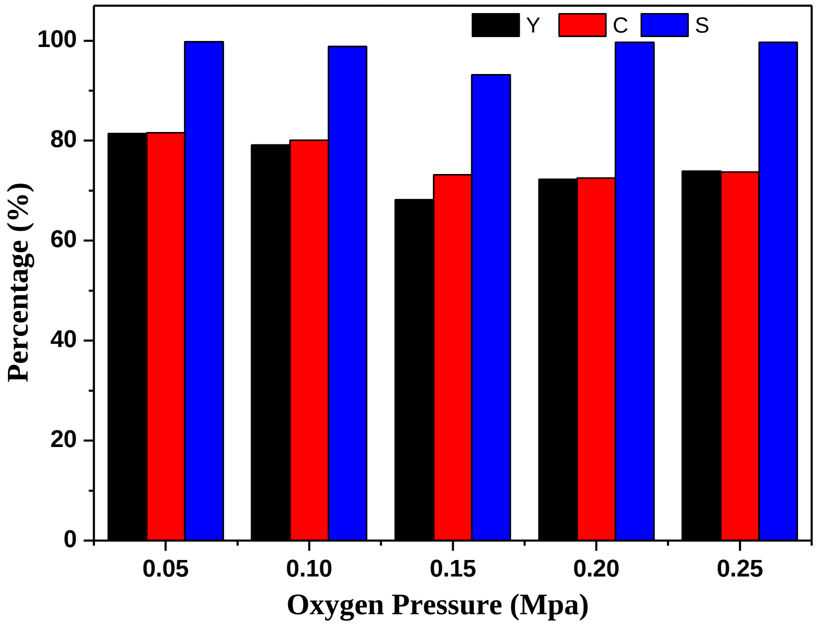

Fig. 4. Effects of the different O_2 pressures on the selective oxidation of benzyl alcohol to benzaldehyde under visible light irradiation (λ>420 nm) for 2 h in O_2 purge condition.

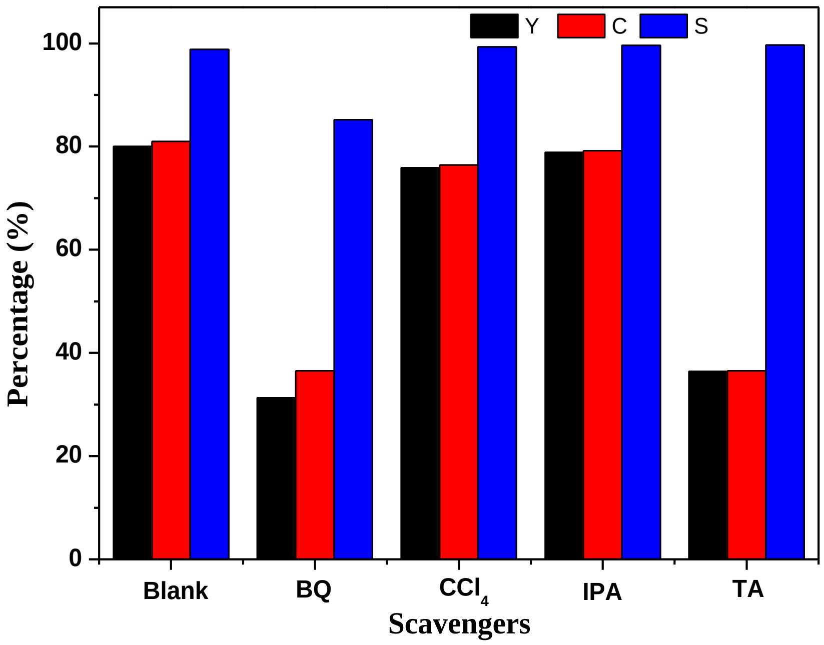

Fig. 5. Photocatalytic performance of adding different additives for selective oxidation of benzyl alcohol to benzaldehyde under visible light irradiation (λ>420 nm) for 2 h in O_2 purge condition.

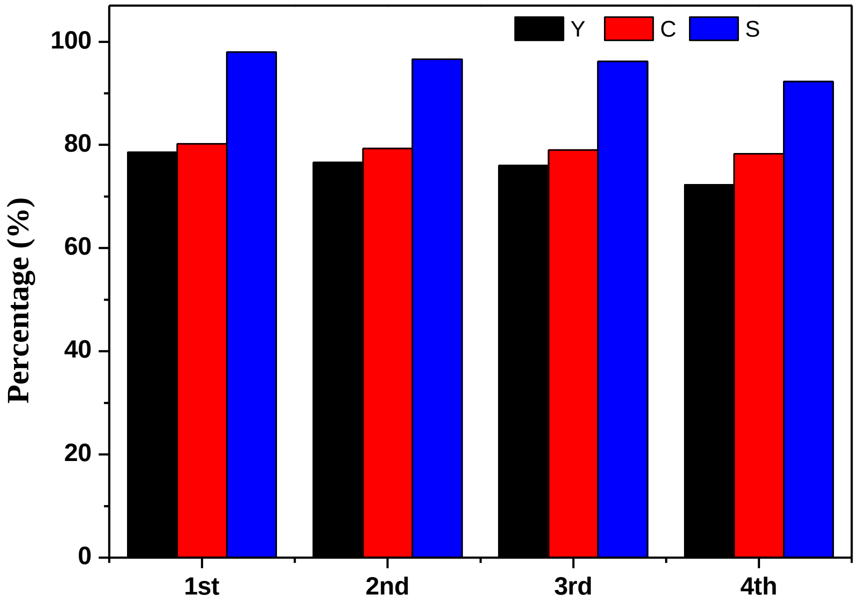

Fig. 6. Photocatalytic performance of recycling testing for selective oxidation of benzyl alcohol to benzaldehyde under visible light irradiation (λ>420 nm) for 2 h in O_2 purge condition.

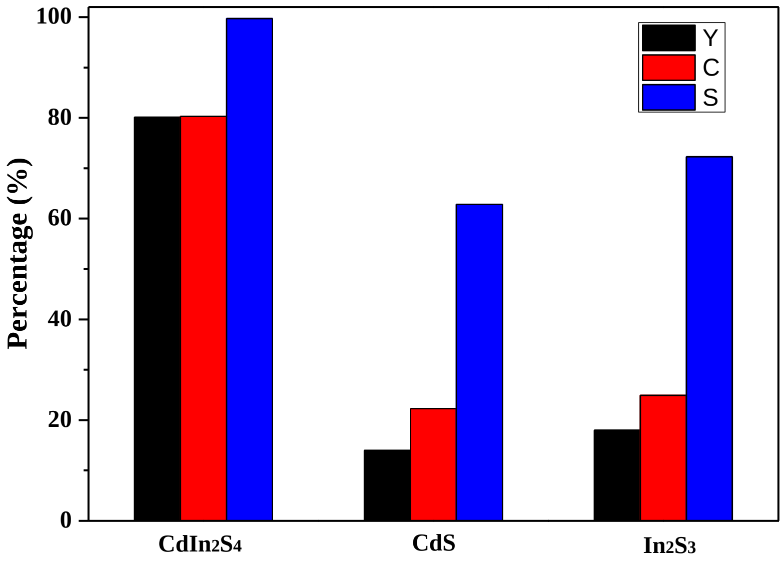

Fig. 7. Photocatalytic performance for selective oxidation of benzyl alcohol to benzaldehyde with CdS, In_2S_3 and $CdIn_2S_4$ photocatalysts under visible light irradiation (λ>420 nm) for 2 h in O_2 purge condition.
